# Effectiveness of a Physical Exercise Prescription Program in Primary Care to Improve Physical Fitness and Quality of Life in Adults Aged 65 Years and Older in a Rural Basic Health Area—Study Protocol for a Randomized Controlled Trial (PREFIS-AP)

**DOI:** 10.3390/sports14100458

**Published:** 2026-10-15

**Authors:** Víctor Manuel González-Rodríguez, Silvia Arroyo-Romero, Esther Martín-López, María Carmela Rodríguez-Martín, Rodrigo Sánchez de Vega-Huidobro, Nuria Sánchez-García, Sergio Campos-Isidro, María Domínguez-Manzano, Luis García-Ortiz, Cristina Lugones-Sánchez

**Affiliations:** 1 Centro de Salud Villoria, Gerencia de Atención Primaria de Salamanca, Gerencia Regional de Salud de Castilla y León (SACYL), Calle Cañada, 2, 37339 Villoria, Spain; silvia_ar@usal.es (S.A.-R.); emartinl@saludcastillayleon.es (E.M.-L.); crodriguezma@saludcastillayleon.es (M.C.R.-M.); rsanchezdv@saludcastillayleon.es (R.S.d.V.-H.); nsanchezga@saludcastillayleon.es (N.S.-G.); scamposi@saludcastillayleon.es (S.C.-I.); mardominguezm@saludcastillayleon.es (M.D.-M.); 2 Instituto de investigación Biomédica de Salamanca (IBSAL), Unidad de Investigación en Atención Primaria de Salamanca (APISAL), Gerencia de Atención Primaria de Salamanca, Gerencia Regional de Salud de Castilla y León (SACYL), Avenida de Portugal 83, 37005 Salamanca, Spain; lgarciao@usal.es (L.G.-O.); clugones@saludcastillayleon.es (C.L.-S.); 3 Departamento de Medicina, Facultad de Medicina, Universidad de Salamanca, Calle Alfonso X el Sabio, s/n, 37007 Salamanca, Spain; 4 Departamento de Ciencias Biomédicas y del Diagnóstico, Facultad de Medicina, Universidad de Salamanca, Calle Alfonso X el Sabio, s/n, 37007 Salamanca, Spain; 5 Departamento de Enfermería y Fisioterapia, Facultad de Enfermería y Fisioterapia, Universidad de Salamanca, Calle Donantes de Sangre, s/n, 37007 Salamanca, Spain

**Keywords:** primary care, exercise prescription, physical activity, older adults, physical fitness, quality of life, randomized controlled trial

## Abstract

Physical inactivity contributes substantially to morbidity, disability, functional decline, and premature mortality in older adults. General physical activity counselling in primary care does not necessarily translate into structured, individualized exercise prescription. PREFIS-AP evaluates a standardized Exercise Prescription Program embedded in routine rural primary care for community-dwelling adults aged 65 years and older. PREFIS-AP is a single-center, pragmatic, parallel-group randomized controlled trial with 210 planned participants allocated 1:1 to a 12-week individualized multicomponent Exercise Prescription Program or usual primary care. The intervention combines clinical and functional assessment, an electronic health record decision-support module, and multicomponent exercise. The primary outcome is the 12-week Six-Minute Walk Test (6MWT) distance adjusted for baseline distance. Secondary outcomes include physical activity, health-related quality of life, strength, lower-limb function, anthropometry, medication burden, and safety; rectus femoris morphology assessed by point-of-care ultrasound is exploratory. The primary analysis will follow the intention-to-treat principle and compare 12-week 6MWT distance between groups adjusted for baseline distance. PREFIS-AP will evaluate the clinical effectiveness and feasibility of integrating structured exercise prescription into routine rural primary care. Limitations include the single-center setting, inability to blind participants and intervention providers, additional exercise-professional support, and 12-week follow-up. Secondary and exploratory analyses are not independently powered.

## 1. Introduction

Population aging is a major public health challenge of the twenty-first century. Although life expectancy continues to increase, additional years of life are frequently accompanied by multimorbidity, functional decline, and disability, placing increasing pressure on health and social care systems. Preserving functional ability, mobility, and independence has therefore become a central objective of the World Health Organization (WHO)’s healthy aging framework [1]. Physical inactivity is an important modifiable determinant of unhealthy aging and is associated with cardiovascular disease, type 2 diabetes, several cancers, disability, and premature mortality [2,3].

Regular exercise improves cardiorespiratory fitness, muscle strength and power, balance, mobility, and health-related quality of life, while contributing to the prevention of falls and functional decline. Progressive resistance training and multicomponent programs combining aerobics, resistance, balance, and functional exercises are particularly relevant for older adults. Current international recommendations therefore support structured, individualized, and appropriately monitored exercise as a core preventive and therapeutic strategy in older populations [3]. This approach is particularly relevant for people at increased risk of frailty and functional decline, for whom multicomponent exercise is an important strategy for preserving functional capacity and independence [3].

Despite this evidence, insufficient physical activity remains highly prevalent, and important socioeconomic and geographical inequalities persist. Rural populations may face specific barriers, including geographical dispersion and reduced availability of opportunities and resources for structured physical activity. These characteristics support the development of pragmatic interventions adapted to and integrated within existing rural primary care structures [4].

Primary care is well placed to deliver such interventions because family physicians and nurses maintain long-term relationships with older adults and routinely assess chronic disease, medication burden, functional status, and other determinants relevant to exercise prescription [5]. However, physical activity promotion in routine clinical practice is often limited to general counseling. Exercise prescription is a more specific clinical process: it translates assessment into an individualized program with defined frequency, intensity, time, type, volume, and progression, alongside appropriate monitoring and adaptation. Limited consultation time, insufficient training, and lack of standardized decision-support tools remain important implementation barriers [4].

Previous Spanish primary care trials provide relevant precedents. PEPAF produced a modest increase in physical activity at 6 months, whereas the available EFICAR publication reports baseline characteristics and treatment optimization rather than comparative intervention outcomes [6,7]. More broadly, a systematic review and meta-analysis of primary care-based physical-activity interventions in adults aged ≥65 years found a small overall increase in physical activity, with substantial heterogeneity across interventions [8]. Multicomponent programs such as Vivifrail have demonstrated improvements in functional capacity in frail older adults; PREFIS-AP differs by embedding individualized exercise prescription within routine primary care and the electronic health record rather than delivering a standalone exercise program [9].

Embedding the prescribing process within the electronic health record may help address some implementation barriers by providing clinicians with a standardized framework for translating clinical and functional information into an individualized exercise prescription. However, when an electronic decision-support system forms part of a multicomponent intervention compared with usual care, its independent contribution cannot be separated from that of the other intervention components. PREFIS-AP therefore evaluates the effectiveness of the complete exercise-prescription strategy rather than the isolated effect of the MEDORA^®^ (electronic health record system; Gerencia Regional de Salud de Castilla y León [SACYL], Valladolid, Spain) decision-support module.

Another limitation of previous research is the relatively limited assessment of objective muscular adaptations. Muscle ultrasound is a portable, non-invasive method for assessing muscle morphology. Ultrasound-detected rectus femoris adaptations have been evaluated after 12 weeks of multicomponent exercise in older adults, although evidence remains limited and standardized acquisition is essential [10,11]. In PREFIS-AP, ultrasound complements emerging primary-care research on muscle assessment in older adults [12,13] but is not intended to diagnose sarcopenia or replace validated functional assessments. Instead, ultrasound-derived measures will be treated as continuous exploratory structural outcomes that may provide complementary information on morphological adaptations and their relationship with changes in physical function.

The PREFIS-AP trial was therefore designed to address these clinical and implementation gaps by combining standardized functional assessment, an individualized multicomponent exercise prescription supported by an electronic health record decision-support module, structured follow-up, point-of-care muscle ultrasound, and predefined fidelity procedures within routine rural primary care.

## 2. Objectives

### 2.1. Primary Objective

The primary objective is to determine whether a 12-week individualized multicomponent Exercise Prescription Program integrated into routine primary care improves functional exercise capacity, assessed as 12-week Six-Minute Walk Test (6MWT) distance adjusted for baseline distance, compared with usual primary care in community-dwelling adults aged 65 years and older.

### 2.2. Secondary Objectives

The secondary objectives are to evaluate the effects of the intervention on physical activity, health-related quality of life, handgrip strength, lower-limb functional performance, anthropometric measures, and medication burden from baseline to 12 weeks.

### 2.3. Exploratory and Implementation Objectives

Exploratory analyses will examine associations between changes in rectus femoris muscle morphology and changes in functional and strength outcomes. A complementary implementation objective is to assess feasibility through recruitment and retention, intervention adherence and fidelity, adverse events, exercise modifications and protocol deviations, reinforcement requirements, and professional contact time. Given the single-center design, these measures will characterize implementation under the conditions of PREFIS-AP rather than establish reproducibility across different healthcare settings.

## 3. Materials and Methods

### 3.1. Study Design

PREFIS-AP is a single-center, pragmatic, parallel-group randomized controlled trial with 1:1 allocation to an individualized Exercise Prescription Program or usual primary care. The protocol follows SPIRIT 2025 recommendations [14], and trial results will be reported in accordance with CONSORT guidance [15]. The SPIRIT checklist is provided as Supplementary Table S1. The trial is registered at ClinicalTrials.gov (NCT07394790). Registration was completed on 6 February 2026. No formal patient or public involvement was planned in the design, conduct, or reporting of the trial.

The overall study pathway is summarized in Figure 1.

### 3.2. Study Setting

The study will be conducted in the primary care setting of the Salamanca Health Area (Castilla y León, Spain). Recruitment will take place at a rural Primary Care Center belonging to the Regional Health Service of Castilla y León. The Basic Health Zone serves 4128 registered users, of whom 35.1% (approximately 1449 people) are aged 65 years or older. The population is geographically dispersed across eight municipalities served by a central Primary Care Center and peripheral local consulting facilities. The zone also includes five residential facilities for older adults, although the trial is restricted to community-dwelling participants.

The project will be coordinated by the Primary Care Research Unit of Salamanca (APISAL) and the Institute for Biomedical Research of Salamanca (IBSAL), both of which have extensive experience in clinical epidemiology, aging research, and exercise prescription.

The intervention will be integrated into routine primary care structures to maximize the pragmatic nature of the trial and facilitate assessment of its potential future implementation within the Spanish National Health System.

### 3.3. Participants and Eligibility Criteria

#### Inclusion and Exclusion Criteria

Eligible participants must fulfill all of the following criteria: age ≥ 65 years; community-dwelling; registered at the participating Primary Care Center; able to walk independently, with or without a walking aid; considered medically suitable for participation in a moderate-intensity multicomponent exercise program; willing to participate; and able to provide written informed consent.

Participants will be excluded when exercise testing or training is considered unsafe according to current pre-participation and exercise-testing standards [16], including myocardial infarction within the previous 3 months; unstable angina; uncontrolled arrhythmia causing symptoms or hemodynamic compromise; symptomatic severe aortic stenosis; decompensated or uncontrolled heart failure; acute pulmonary embolism or acute deep-vein thrombosis; uncontrolled respiratory disease or acute respiratory failure; acute systemic illness likely to impair exercise capacity or worsen with exercise; established dementia or an ongoing diagnostic process for suspected dementia when reliable consent or study participation cannot be ensured; severe psychiatric illness preventing cooperation with the intervention or assessments; or any other clinical condition that, in the investigating family physician’s judgment, could compromise participant safety.

Participants who routinely use a walking aid will be eligible and will use the same aid during the 6MWT and throughout study-related exercise, as required. Previous participation in structured exercise or regular physical activity is not an exclusion criterion, consistent with the trial’s real-world effectiveness objective. The study therefore evaluates an older primary care population rather than a population defined exclusively by sedentary behavior.

Potential participants will be identified and invited by their Primary Care professionals. Medical suitability will be determined through a review of the electronic health record, medical history, current medication, relevant comorbidities, symptoms, and functional status, together with a focused clinical examination when indicated. The family physician will also assess participants’ capacity to understand the study, appreciate its implications, and provide informed consent.

### 3.4. Recruitment

Recruitment will be promoted across all consulting facilities in the Basic Health Zone to reduce discretionary selection and dependence on attendance at a single consultation. Potentially eligible adults will be identified and invited consecutively by primary care professionals when encountered during routine care. All interested individuals will receive verbal and written information describing the study objectives, procedures, potential risks, and expected benefits. Written informed consent will be obtained by a member of the research team before any study-specific assessment, and baseline assessments will be completed before randomization.

The study will be conducted in a rural Primary Care Center serving eight municipalities, with a source population of approximately 1449 registered adults aged 65 years or older. Although the trial is conducted within a single Primary Care Center, this population is geographically distributed across the eight municipalities served by the center. Recruitment will involve the primary care professionals serving these municipalities, thereby allowing potentially eligible participants to be identified across the entire catchment population rather than from a single practice. Recruitment records will distinguish individuals approached, individuals assessed for eligibility, exclusions, refusals, and participants ultimately randomized. The planned sample comprises 210 randomized participants, with 105 allocated to each study group, representing approximately 14.5% of the population aged 65 years or older registered in the Basic Health Zone.

### 3.5. Randomization and Allocation Concealment

After completion of the baseline assessment, participants will be allocated 1:1 using simple unrestricted randomisation implemented in REDCap, without blocking or stratification, by an independent researcher. The investigator conducting the baseline assessment will initiate the randomization procedure but will have no ability to predict, alter, or override the allocation. Allocation will remain concealed until the participant has been enrolled and the baseline assessment has been completed; REDCap will then electronically reveal the group assignment, independently of participant characteristics. The same investigator will perform the baseline and 12-week assessments and, having initiated the randomization procedure, will therefore be aware of group allocation at follow-up. Standardized assessment procedures will be used to minimize measurement bias.

The investigator responsible for the baseline assessment will not be involved in participant recruitment or delivery of the exercise intervention. Due to the nature of the intervention, participants and professionals delivering the individualized Exercise Prescription Program cannot be blinded to group allocation. Outcome assessment procedures will be standardized to minimize measurement bias. The exercise professional delivering the intervention will not be involved in outcome assessments or statistical analysis. The statistical analyst will be blinded to group allocation through coded group labels during the primary analysis.

### 3.6. Study Assessments

All participants will undergo the same assessment battery at baseline and at 12 weeks after randomization and intervention initiation (Table 1).

Baseline assessments will be completed before randomization. Assessments will be performed by trained investigators following standardized operating procedures, with an independent investigator overseeing quality assurance. Whenever possible, the same equipment, procedures, testing conditions, and measurement environment will be used at both time points. The assessment battery comprises sociodemographic and clinical characteristics, medication use, anthropometry and blood pressure, laboratory variables, physical activity, health-related quality of life, functional exercise capacity, muscle strength, lower-limb functional performance, and muscle morphology.

#### 3.6.1. Sociodemographic and Clinical Assessment

Age, sex, marital status, educational attainment, and occupational status will be recorded at inclusion. Medical history, chronic diseases, and medication use will be obtained from the electronic health record and verified during the study visit.

Medication burden will be operationalized as the number of regularly prescribed medications recorded in the electronic health record at each assessment. Changes in medication burden will be considered a secondary outcome, while medication-related analyses beyond this predefined measure will be considered exploratory.

#### 3.6.2. Anthropometric and Clinical Examination

Body weight will be measured twice using a calibrated Seca 770 electronic scale (accuracy ± 0.1 kg), and height using a Seca 222 stadiometer, with the participant barefoot and standing upright. The mean of two measurements will be recorded. Body mass index will be calculated as kg/m^2^. Waist circumference will be measured using a non-elastic tape according to SEEDO recommendations [17], and calf circumference will be measured at the point of maximum circumference. Calf circumference will be considered an anthropometric indicator related to peripheral muscle quantity but will not be used in isolation to diagnose sarcopenia [18].

Resting blood pressure will be measured with an automated OMRON M10 device after at least 5 min of seated rest. Three measurements will be taken, and the mean of the last two will be used for analysis, following European recommendations [19].

#### 3.6.3. Laboratory Assessment

Fasting venous blood and urine samples will be collected in the morning using standardized pre-analytical procedures. Routine biochemical, hematological, renal, metabolic, and inflammatory parameters, together with the urine albumin-to-creatinine ratio, will be analyzed in the reference hospital laboratory using standardized procedures. These laboratory variables will be considered descriptive or exploratory rather than confirmatory efficacy outcomes.

#### 3.6.4. Physical Activity and Health-Related Quality of Life

Physical activity will be assessed using the GPAQ and scored according to the WHO GPAQ Analysis Guide [20,21]. The standardized scoring procedure will be applied to derive the corresponding physical activity indicators, covering work, transport, recreational activity, and sedentary behavior. The same questionnaire and administration procedure will be used at baseline and at the 12-week follow-up.

Health-related quality of life will be assessed using the validated Spanish version of the 36-Item Short Form Health Survey (SF-36), including its eight health domains and physical and mental component summary scores, calculated according to the validated scoring procedure [22,23].

#### 3.6.5. Physical Performance

Functional exercise capacity will be assessed using the Six-Minute Walk Test (6MWT), the primary outcome measure. Participants will be instructed to walk as far as possible for six minutes along a standardized 20-m level walkway, turning around the markers at each end. No practice walk will be performed. Standardized instructions and predefined encouragement phrases will be used at one-minute intervals; if a participant stops, standardized encouragement to resume walking will be provided. Total distance walked will be recorded in meters. Heart rate, oxygen saturation, blood pressure, and perceived exertion measured using the Borg scale will be monitored according to the standardized operating procedure [24]. Participants who routinely use a walking aid will use the same aid at baseline and follow-up.

The same course, instructions, and testing conditions will be maintained at both assessments. Because walkway length can influence absolute 6MWT distance through differences in the number of turns, the 20-m course is explicitly reported and should not be considered equivalent to the conventional 30-m course. Although derived from patients with COPD, available evidence suggests that shorter standardized courses may be used for repeated assessments when course length and testing procedures are consistently maintained [25]. The primary endpoint will be the between-group difference in 6MWT distance at 12 weeks adjusted for baseline 6MWT distance.

Upper-limb muscle strength will be assessed bilaterally using an analog JAMAR handgrip dynamometer and standardized positioning, with participants seated, the shoulder adducted, elbow flexed to approximately 90°, forearm in a neutral position, and wrist in a comfortable functional position. Standardized verbal instructions will be provided and three trials will be performed with each hand, with 15 s of rest between trials. The highest value for each hand will be retained, and the analyzed value will be the sum of the highest values obtained for the right and left hands. The same dynamometer and testing procedure will be used at baseline and follow-up.

Lower-limb functional performance will be assessed using the 30-s Sit-to-Stand Test. Participants will rise completely from a standardized chair and return to the seated position as many times as possible within 30 s, with their arms crossed over the chest unless a safety-related adaptation is required. The number of correctly completed chair rises will be recorded [26]. The test will be considered a measure of lower-limb functional performance rather than a direct measure of isolated muscle strength. A specific seat height and a practice trial were not prespecified in the study operating procedure.

#### 3.6.6. Muscle Morphology

Rectus femoris morphology will be assessed using an ESAOTE MyLab50XVision ultrasound device (ESAOTE S.p.A., Firenze, Italy), using a high-resolution, 5–10 MHz multifrequency linear transducer as a complementary structural assessment of skeletal muscle. Participants will be examined in the supine position with their lower limbs relaxed. The transducer will be positioned transversely at predefined anatomical landmarks along the line between the anterior superior iliac spine and the superior pole of the patella. Images will be obtained at standardized locations, at the midpoint and distal third of the right thigh, using the same acquisition protocol at baseline and follow-up. Sufficient ultrasound gel will be used to minimize tissue compression, and excessive probe pressure will be avoided. Acquisition settings and participant positioning will be kept as consistent as possible between assessments [13].

Rectus femoris muscle thickness will be analyzed as a continuous morphological outcome and will not be used to establish a study diagnosis of sarcopenia [27,28]. Ultrasound assessment will be considered exploratory and is intended to examine whether changes in muscle morphology accompany changes in strength and functional performance.

Exploratory ultrasound outcomes will be rectus femoris thickness (mm) measured at the midpoint and distal third of the right thigh. Ultrasound acquisition and measurements will be performed at baseline and 12 weeks by the same investigator to minimize inter-observer variability, with no subsequent offline image-reading phase planned. Device settings, image-processing procedures, and formal intra- or inter-observer reliability assessments were not prespecified before enrolment.

### 3.7. Sample Size Calculation

The sample size was calculated a priori using G*Power version 3.1 [29]. The sample-size calculation was based on the between-group difference in change in 6MWT distance from baseline to 12 weeks. A clinically relevant difference of 26 m and an SD of 60 m for the change scores were assumed, corresponding to a standardized effect size (Cohen’s d) of 0.433. A change of approximately 20 m has been identified as a small meaningful change in 6MWD in older adults [30,31]. With a two-sided α of 0.05 and 80% power, 85 participants per group are required. Allowing for approximately 19% attrition, the final target sample was set at 105 participants per group (210 participants overall). The calculation was based on an unadjusted between-group comparison; the primary analysis will use baseline-adjusted 12-week 6MWT distance. The trial is powered for the primary outcome, not for secondary or exploratory outcomes.

### 3.8. Intervention

Exercise will be individually prescribed and performed both independently at home and during supervised group sessions available three days per week in municipal sports facilities. During supervised sessions, each participant will follow his or her individualized prescription under the supervision of the exercise professional. These sessions will provide reinforcement, supervision of exercise performance, and adaptation of the prescription when required throughout the 12-week intervention. Initial exercise dose will be individualized according to baseline clinical and functional assessment within the prescription ranges described below.

Participants allocated to the intervention group will receive a 12-week individualized multicomponent Exercise Prescription Program integrated into routine primary care [3,32,33]. Prescriptions will be generated using the exercise-prescription module embedded in MEDORA^®^, the regional primary care electronic health record, through predefined clinical algorithms informed by baseline clinical and functional assessment [34]. These algorithms will support prescription while allowing modification according to clinical judgment, participant characteristics, tolerance, and safety. The intervention will be described according to the TIDieR framework [35] and will follow the FITT-VP principles of frequency, intensity, time, type, volume, and progression [3]. Table S2.

#### 3.8.1. Exercise Components and Progression

Aerobic exercise. Activities may include walking, cycling, swimming, and comparable activities, adapted to baseline functional capacity, clinical status, accessibility, and participant preference. Intensity will be individualized using perceived exertion, including the Borg Rating of Perceived Exertion Scale [36]. Depending on baseline fitness, tolerance, and functional response, participants may progress from intermittent to continuous exercise and, when appropriate, to interval training. Warm-up and cool-down periods will form part of the prescribed sessions.

Resistance exercise. Progressive resistance exercise will be performed primarily using elastic bands and functional resistance exercises. Resistance, repetitions, sets, and recovery will be individualized, with perceived exertion supported by the OMNI-RES scale [37]. Progression may involve modification of resistance, repetitions, sets, exercise complexity, or recovery according to tolerance, perceived exertion, functional response, safety, and correct execution.

Balance exercise. Balance training will include progressively challenging static and dynamic tasks, including tandem stance or walking, single-leg stance when safe, multidirectional stepping, and gait-related activities. Difficulty will be individualized and progressed according to the participant’s functional capacity, safety, and ability to perform the preceding level correctly.

Flexibility. Flexibility exercises will target major muscle groups and will be incorporated into the individualized program and, where appropriate, into warm-up and cool-down periods.

Inspiratory muscle training. Inspiratory muscle training will not be prescribed routinely to all participants but only when clinically indicated within the individualized prescription. Inspiratory muscle training will be included when it is considered clinically indicated; specific eligibility criteria and a standardized dose will not be prespecified before enrolment. When required for prescription, maximal inspiratory pressure will be obtained from information available in the participant’s clinical record and will not be measured as a study-specific outcome. When prescribed, it will be performed using a threshold inspiratory device, with intensity individualized according to maximal inspiratory pressure and progressively adapted according to participant tolerance and clinical response.

Individualization and progression. Frequency, intensity, time, type, volume, and progression will be individualized within the FITT-VP framework according to baseline functional capacity, adherence, perceived exertion, tolerance, functional response, and safety [3]. One or more FITT-VP components may therefore be modified during follow-up. Although individual prescriptions may differ between participants, the assessment, decision-making, and prescribing process will remain standardized.

#### 3.8.2. MEDORA^®^ Decision-Support Process

The MEDORA^®^ exercise-prescription module will support clinical decision-making by integrating baseline clinical suitability, functional capacity, physical performance, relevant comorbidities, medication-related considerations, and individual participant characteristics [34]. Medication-related information will inform exercise safety and individualization; medication review or modification is not part of the intervention. The module will assist the healthcare professional in generating an individualized multicomponent exercise prescription but will not replace clinical judgment. The exercise prescription generated through the MEDORA^®^ pathway will be formally validated by the principal investigator.

PREFIS-AP evaluates the complete intervention strategy, including clinical and functional assessment, MEDORA^®^-supported prescription, written instructions, exercise-professional support, reinforcement, and follow-up. Consequently, the trial is designed to estimate the effect of the overall Exercise Prescription Program compared with usual care, rather than the independent causal effect of the MEDORA^®^ module.

#### 3.8.3. Support, Adherence, and Intervention Fidelity

Participants will receive verbal and written instructions before beginning the program. A qualified exercise professional will support correct performance, safe progression, and adherence in the intervention group. Approximately two weeks after program initiation, a reinforcement contact will be conducted to verify understanding, identify barriers, assess early adherence, review progression, and identify adverse events or incidents.

Adherence will be prospectively assessed using participant exercise records, scheduled contacts, and available attendance information. Adherence will be defined as the percentage of prescribed exercise sessions completed. Exercise modifications, interruptions, protocol deviations, and adverse events will be documented. Intervention fidelity will be promoted through standardized investigator training, operating procedures, predefined algorithms within MEDORA^®^, standardized assessment materials, regular investigator meetings, and prospective documentation of protocol deviations and exercise modifications. Although exercise prescriptions will be individualized, the prescribing process itself will be standardized.

### 3.9. Control Group

Participants allocated to the control group will receive usual primary care, including non-standardized general physical-activity advice consistent with current WHO recommendations [33], rather than a study-specific counselling intervention. They will not receive the individualized exercise prescription generated through the PREFIS-AP MEDORA^®^ module, structured intervention materials, or the individualized multicomponent Exercise Prescription Program during the 12-week study period. They also will not receive any other individualized instructions. Participants in both groups will continue to receive any medical care considered necessary.

Because participants live within the same rural communities, complete prevention of exposure to independent community physical activity resources is not possible. Such co-interventions, together with changes in physical activity assessed during follow-up, will therefore be considered when interpreting the findings.

### 3.10. Outcome Measures

Primary outcome. The primary outcome is functional exercise capacity, assessed as 6MWT distance in meters [38]. The primary treatment effect will be the between-group difference in 6MWT distance at 12 weeks adjusted for baseline 6MWT distance. The clinically relevant difference of 26 m used for sample-size planning is not part of the outcome definition [30].

Secondary outcomes. Secondary outcomes include changes from baseline to 12 weeks in GPAQ-derived physical activity; SF-36 physical and mental component summary scores; handgrip strength; 30-s Sit-to-Stand performance; anthropometric measures (weight, height, body mass index, waist circumference, hip circumference, and calf circumference); medication burden; and intervention safety. The eight individual SF-36 domain scores will be considered exploratory. Implementation outcomes will include recruitment and retention, intervention adherence and fidelity, adverse events, exercise modifications and protocol deviations, reinforcement requirements, and professional contact time; these outcomes will be analyzed descriptively. Associations between changes in ultrasound-derived muscle morphology and changes in functional and strength outcomes will be considered exploratory. Laboratory variables will also be considered descriptive or exploratory rather than confirmatory efficacy outcomes.

### 3.11. Data Collection and Management

Data will be recorded in standardized electronic case report forms and stored in a secure electronic database with predefined range and consistency checks. Participants will be identified by unique study codes; identifying information and the re-identification key will be stored separately from the analytical dataset, with access restricted to authorized members of the research team. Data quality will be reviewed periodically, and all corrections will be documented and traceable.

The analytical database will contain coded treatment groups, with access restricted according to predefined study responsibilities. The schedule of enrolment, interventions, and assessments is summarized in Figure 2.

### 3.12. Statistical Analysis

Continuous variables will be summarized as the mean (standard deviation) or median (interquartile range), as appropriate, and categorical variables as frequencies and percentages. Baseline characteristics will be described by randomized group without significance testing as a criterion of successful randomization.

The primary analysis will follow the intention-to-treat principle, with participants analyzed according to their randomized group. The primary treatment effect will be estimated using a baseline-adjusted regression model equivalent to analysis of covariance (ANCOVA), with 12-week 6MWT distance as the dependent variable, randomized group as the principal explanatory variable, and baseline 6MWT distance as a covariate. No additional covariates will be included in the primary model. The intervention effect will be reported as an adjusted between-group mean difference with a 95% confidence interval and two-sided *p*-value. Missing 12-week outcome data will be addressed by multiple imputation as a sensitivity analysis. A per-protocol sensitivity analysis will include participants completing ≥70% of prescribed exercise sessions; adherence will be calculated as the number of completed sessions divided by the number prescribed during the 12-week intervention, multiplied by 100.

Secondary continuous outcomes will be analyzed using analogous baseline-adjusted models, and appropriate generalized regression models will be used for categorical outcomes. Secondary and structure–function analyses will be considered exploratory. No adjustment for multiplicity will be applied to secondary outcomes, and no confirmatory claims will be based on these analyses; interpretation will emphasize effect estimates and 95% confidence intervals.

The amount and pattern of missing outcome data will be examined. The primary analysis will be a complete-case analysis, assuming data are missing at random conditional on the model covariates. Multiple imputation will be performed as an unconditional sensitivity analysis using 20 imputations and including treatment allocation, baseline and 12-week 6MWT distance, age, and sex.

All statistical tests will be two-sided, with α = 0.05 for the primary hypothesis. No interim efficacy analysis or formal statistical stopping rule is planned. A detailed statistical analysis plan will be finalized before database lock and before unblinded examination of between-group outcome comparisons.

The finalized Statistical Analysis Plan (SAP) was deposited on Zenodo on 29 September 2026 (https://doi.org/10.5281/zenodo.23034729), before any outcome-data analysis. It distinguishes prospectively established procedures from analytical specifications finalized after data collection.

### 3.13. Safety and Adverse Events

Safety monitoring will cover the intervention and assessment period from enrolment until completion of the 12-week follow-up. An adverse event will be defined as any unfavorable medical occurrence during study participation, whether or not considered related to the intervention. An intervention-related adverse event will be any event for which a reasonable temporal and clinical relationship with exercise testing or prescribed exercise cannot be excluded. Events of specific interest include falls, musculoskeletal pain or injury, dizziness, syncope or presyncope, exercise intolerance, cardiovascular or respiratory exacerbation, and any symptom requiring interruption or modification of exercise.

Participants will be instructed to report symptoms or health problems and to stop exercise and contact the study team if warning symptoms occur. Adverse events will also be actively assessed during scheduled contacts. For every event, investigators will document its nature, onset, severity, clinical course, action taken, outcome, and possible relationship to the study procedures.

A serious adverse event will be defined as an event resulting in death, posing an immediate threat to life, requiring or prolonging hospitalization, causing persistent or clinically significant disability, or otherwise judged medically important. An exercise-related injury will be defined as a musculoskeletal complaint or injury occurring during or following prescribed exercise that requires modification or temporary interruption of exercise, medical assessment, or treatment.

Temporary suspension or modification of the exercise prescription will be considered in the event of acute illness, clinically relevant pain or injury, new cardiovascular or respiratory symptoms, or any condition requiring clinical reassessment. Permanent discontinuation of the exercise intervention may be decided by the investigating physician when continued participation is considered unsafe, without withdrawing the participant from outcome follow-up whenever feasible. Participants will retain access to routine clinical and emergency care through the Primary Care Center’s usual procedures.

Safety oversight will be performed by the physician principal investigator together with the collaborating clinical investigators. Serious or unexpected events considered related to study procedures will be promptly communicated to the principal investigator and reported to the Research Ethics Committee in accordance with applicable requirements. A separate Data Monitoring Committee is not planned because the intervention is short, non-pharmacological, and considered low risk, with direct medical oversight. No formal statistical stopping rule or interim efficacy analysis is planned.

### 3.14. Ethics and Dissemination

The study was approved by the Research Ethics Committee of the Salamanca Health Area (Ref. CEIm: PI 2024 05 1615); the favorable ethical opinion was issued on 27 May 2024. The trial will be conducted in accordance with the Declaration of Helsinki [39], International Council for Harmonisation Good Clinical Practice (ICH-GCP) principles [40], and applicable European and Spanish legislation concerning biomedical research and personal-data protection.

Written informed consent will be obtained from every participant before any study-specific procedure. Substantive protocol amendments affecting participant safety, study procedures, outcomes, or scientific integrity will require ethics approval and updating of the public trial record, as appropriate.

Study findings will be disseminated through scientific publications and presentations irrespective of the direction or statistical significance of the results.

### 3.15. Trial Status

The current manuscript reflects the revised PREFIS-AP protocol, September 2026. The trial was prospectively registered at ClinicalTrials.gov (NCT07394790): the study was first submitted on 12 December 2025, the first submission meeting quality-control criteria was received on 5 February 2026, and the record was first posted on 6 February 2026. Participant recruitment began after the trial had been publicly registered and took place during the first half of 2026. The 12-week intervention period and post-intervention assessments have been completed. The last participant was randomized on 7 May 2026, and the final 12-week assessment was completed on 2 July 2026. At the time of this protocol revision, no outcome data have been cleaned, modified, or analysed, either pooled or by treatment group. The finalized, date-stamped SAP is publicly available on Zenodo (https://doi.org/10.5281/zenodo.23034729). This manuscript reports the study protocol and does not present comparative trial results.

### 3.16. Use of Artificial Intelligence-Assisted Technologies

During the preparation of this manuscript, the authors used artificial intelligence-assisted tools, including ChatGPT (OpenAI), to support literature searches, explore relevant scientific evidence, organize preliminary ideas, provide language-editing support, and assist in the preliminary design and refinement of graphical materials. These tools were used solely as complementary aids and are not considered sources of scientific evidence in themselves. No artificial intelligence tool was used to generate, modify, analyze, or interpret study data. All retrieved information and references were critically reviewed and verified by the authors against the original scientific sources before inclusion in the manuscript. All scientific content and graphical elements were reviewed and approved by the authors, who take full responsibility for the integrity and accuracy of the final manuscript.

## 4. Discussion

The challenge is no longer simply to demonstrate that exercise benefits older adults but to identify effective strategies for incorporating exercise prescription into routine primary care. PREFIS-AP addresses this implementation gap by evaluating a standardized yet individualized program embedded in the electronic health record rather than a conventional standalone community exercise program. The model combines objective functional assessment, algorithm-supported clinical decision-making, individualized multicomponent exercise, and structured follow-up while preserving individualized care.

An important feature of PREFIS-AP is the distinction between general physical activity counseling and individualized exercise prescription. The intervention translates clinical and functional assessment into a structured multicomponent program based on the FITT-VP framework [41], incorporating individualized progression, monitoring, and follow-up. A further strength is the integration of individualized exercise prescription into routine Primary Care through the MEDORA^®^ electronic health record, facilitating its incorporation into the usual clinical workflow. The intervention is supported by a structured clinical exercise-prescription framework that defines exercise components, prescription ranges, and progression principles according to baseline clinical and functional assessment. Any observed treatment effect will therefore reflect the combined contribution of individualized prescription, electronic decision support, written instructions, exercise-professional support, reinforcement, progression, and follow-up; the study is not designed to determine the independent effectiveness of the electronic decision-support module.

A major strength of this study is the multimodal assessment of muscle health. Alongside validated measures of functional capacity, muscle strength, physical activity, and health-related quality of life, the protocol incorporates point-of-care ultrasound of the rectus femoris as an objective continuous measure of muscle morphology [13]. Ultrasound is increasingly recognized as a promising, portable, and non-invasive tool for muscle assessment, but standardization remains incomplete [41]. Methodological heterogeneity, the absence of universally accepted diagnostic thresholds, and potential measurement variability should therefore be considered when interpreting longitudinal changes. Ultrasound-derived measures in PREFIS-AP will consequently be treated as exploratory structural outcomes rather than diagnostic criteria for sarcopenia. Their inclusion may provide complementary information on morphological adaptations and their relationship with changes in strength and functional performance.

The intervention was also designed to promote consistency in the prescribing process. Exercise prescription follows current international recommendations and the FITT-VP framework [41], while structured procedures and fidelity monitoring are intended to reduce avoidable variation between professionals [3]. Its delivery through existing primary care structures increases clinical relevance. Recruitment, clinical assessment, and prescription are integrated into routine care, and previous participation in regular physical activity is not an exclusion criterion, consistent with the pragmatic nature of the trial. Nevertheless, recruitment through routine primary care, willingness to participate, independent ambulation, and capacity to provide informed consent may result in the selection of a healthier or more engaged population and should be considered when interpreting the generalizability of the findings.

Several limitations should be acknowledged. Blinding of participants and professionals delivering the intervention is not feasible because of the behavioral nature of the intervention. Participant awareness of group allocation may influence self-reported outcomes, particularly GPAQ-derived physical activity and SF-36 health-related quality of life, through expectations or reporting behavior. The GPAQ has not been specifically validated in a comparable population of Spanish older adults. Follow-up outcome assessors cannot be assumed to remain fully blinded to group allocation; standardized assessment procedures and separation of intervention delivery from outcome assessment are intended to reduce measurement bias, while the statistical analyst will remain blinded to group identity through coded treatment labels. Predefined criteria and dosing for inspiratory muscle training and technical settings and formal reliability assessment for ultrasound were not prespecified before enrolment; the latter should be considered when interpreting small ultrasound changes over 12 weeks.

The complete internal decision logic of the MEDORA^®^ exercise-prescription module, including all input variables, decision thresholds, and output rules, is not publicly available. Therefore, although the clinical exercise-prescription framework, permitted prescription ranges, tailoring principles, and intervention delivery procedures are described, the electronic decision-support component itself cannot be independently reproduced from the information reported in this protocol. Accordingly, the present trial evaluates the complete exercise-prescription strategy implemented in Primary Care rather than the isolated effect of the MEDORA^®^ module.

In addition, recruitment is limited to a single rural Primary Care Center, which may reduce generalizability to urban primary care populations or healthcare settings with different demographic and organizational characteristics. The geographically dispersed catchment population provides an appropriate setting in which to evaluate the intervention under routine rural primary care conditions, but it should not be assumed to be representative of all rural communities. Future multicenter studies, including urban settings, will therefore be required to assess transferability to other healthcare contexts. Furthermore, the additional support of a qualified exercise professional in the intervention arm represents an important departure from usual care and should be considered when judging scalability [5]. Future implementation research should examine the personnel, time, organizational, and economic resources required for broader implementation.

Participants in both study groups live within the same rural communities and may have access to common walking routes, municipal facilities, or other physical activity resources. Contamination and co-interventions therefore cannot be completely excluded and should be considered when interpreting the findings. The 12-week follow-up allows assessment of short-term functional response but not the long-term sustainability of exercise adherence or treatment effects, highlighting the need for future longitudinal research. Finally, the trial is powered specifically for the 6MWT primary outcome and not for all secondary or exploratory outcomes. Secondary analyses should therefore be interpreted primarily in terms of effect estimates, confidence intervals, and consistency of findings, while ultrasound-derived measures and structure-function associations should be considered exploratory and hypothesis-generating.

Despite these limitations, several features strengthen the transparency and methodological rigor of this study, including prospective registration, SPIRIT-informed protocol reporting, standardized operating procedures, concealed electronic allocation, standardized clinical and functional assessment, individualized multicomponent exercise prescription, electronic-health-record decision support, structured reinforcement, intervention fidelity monitoring, and systematic safety monitoring [14,15]. The explicit description of the setting, intervention components, decision-support process, adherence assessment, and fidelity procedures should also facilitate external appraisal and future replication.

If effective, PREFIS-AP could provide a framework for moving from general physical activity counseling to structured exercise prescription in primary care. Beyond estimating the clinical effectiveness of the complete intervention strategy, the trial can contribute to the structured prescribing framework and its integration into the electronic health record, which can make consistent implementation within Primary Care easier. Further multicenter studies with longer follow-up and specific implementation and economic outcomes will be required to determine its broader effectiveness, sustainability, transferability, and scalability.

## Figures and Tables

**Figure 1:**
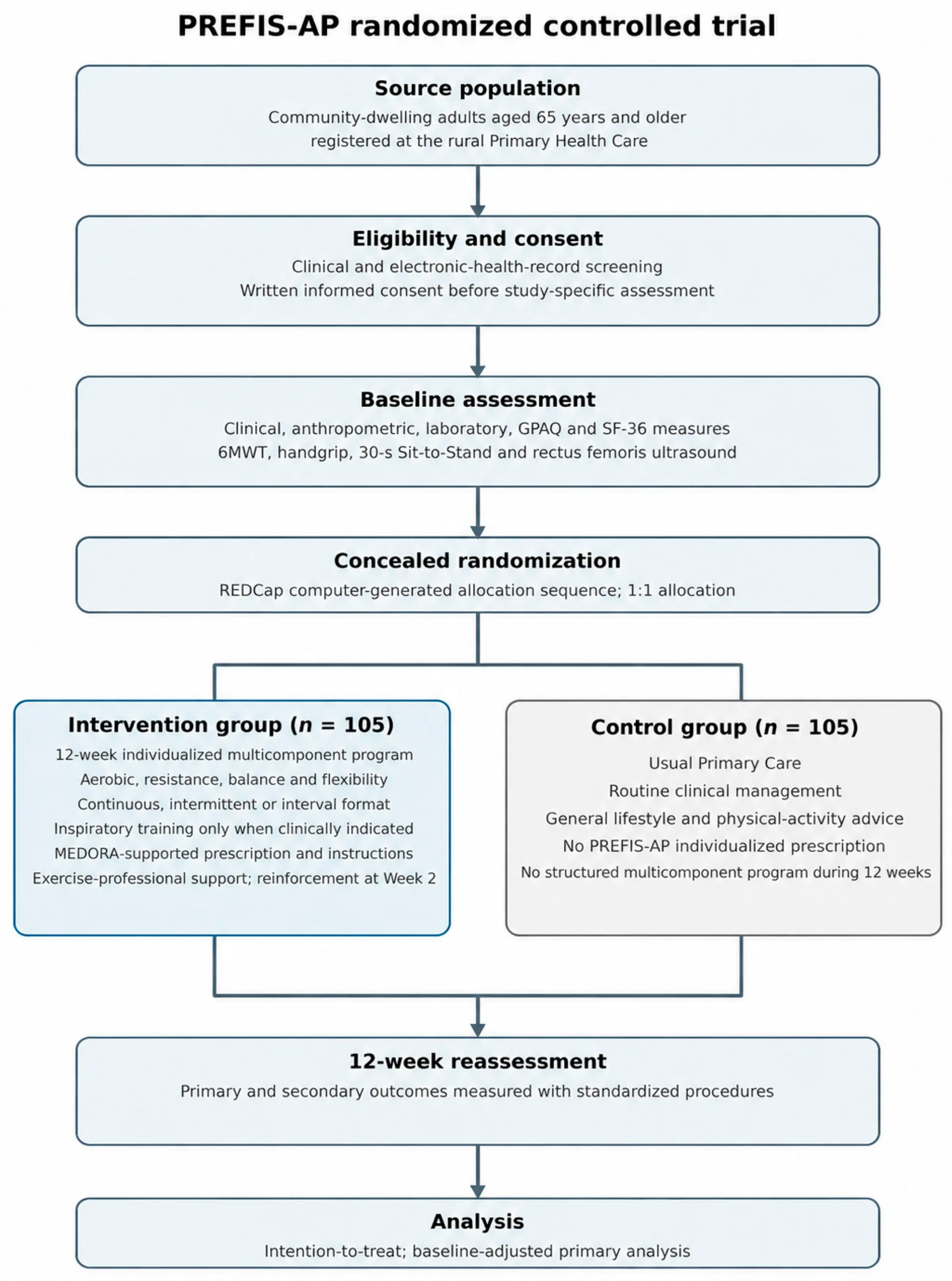
Study flow diagram of the PREFIS-AP randomized controlled trial.

**Figure 2:**
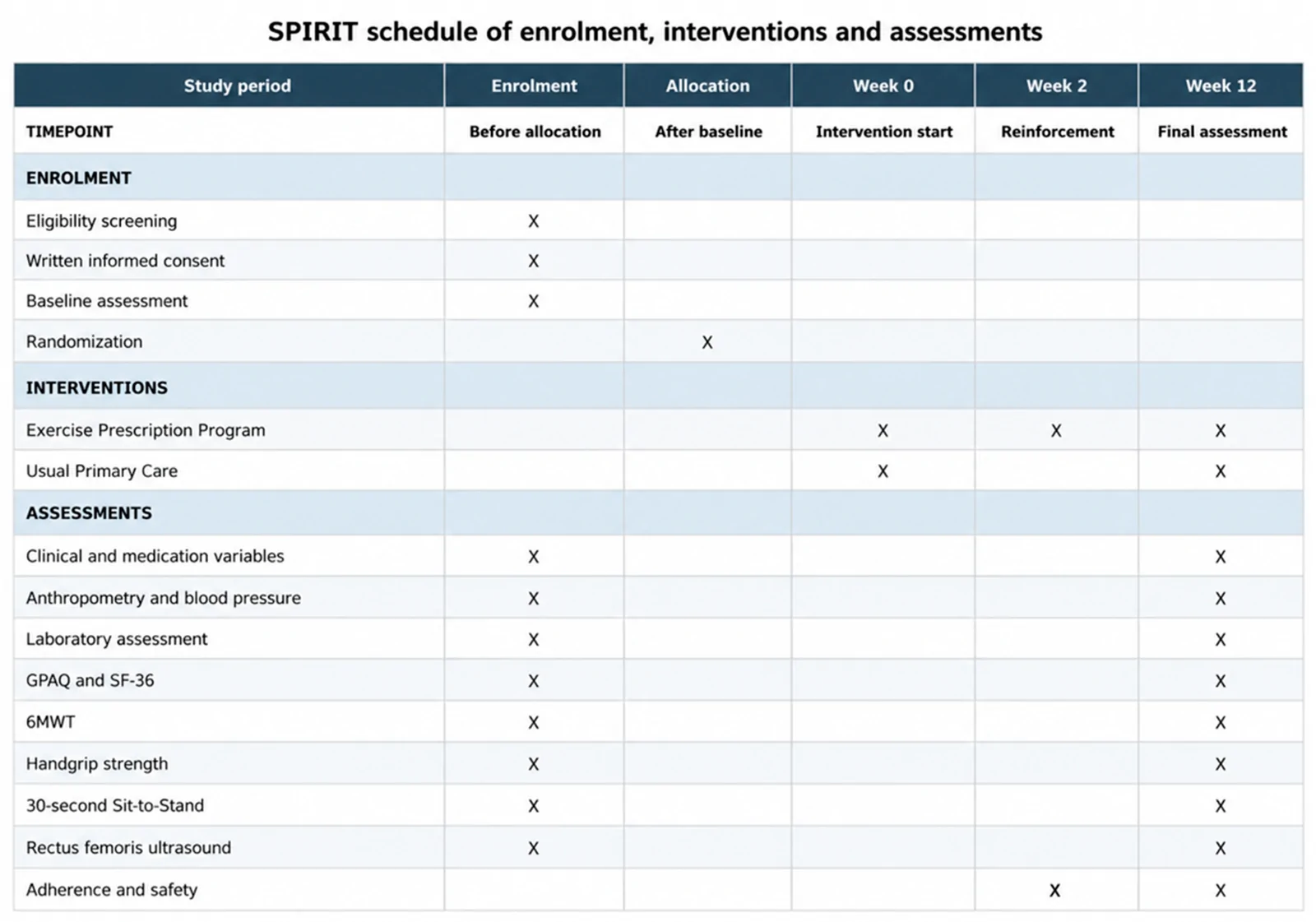
SPIRIT 2025 schedule of enrolment, interventions and assessments for the PREFIS-AP randomized controlled trial.

**Table 1:** Summary of study assessments.

Assessment Domain	Instrument/Method	Main Outcome
Sociodemographic/clinical	Structured interview and electronic health record	Demographic and clinical characteristics
Anthropometry	Calibrated scale, stadiometer and measuring tape	BMI; waist and calf circumference
Blood pressure	Automated sphygmomanometer	Systolic and diastolic blood pressure
Laboratory	Blood and urine analyses	Metabolic, renal, hematological and inflammatory parameters
Physical activity	GPAQ	Physical activity and sedentary behavior
Quality of life	SF-36	Domain and summary scores
Functional exercise capacity	6MWT	Distance walked (m)—primary outcome
Upper-limb strength	JAMAR dynamometer	Handgrip strength (kg)
Lower-limb performance	30 s Sit-to-Stand Test	Number of complete repetitions
Muscle morphology	Rectus femoris ultrasound	Ultrasound-derived muscle measures
Safety	Structured adverse-event reporting	Intervention-related adverse events

Abbreviations: BMI: body mass index; GPAQ: Global Physical Activity Questionnaire; SF-36: 36-Item Short Form; 6MWT: Six-Minute Walk Test.

## Data Availability

The datasets generated during the study will be deposited in the IBSAL community on Zenodo repository (DOI: 10.5281/zenodo.23034729). Anonymized data will be available through a controlled-access procedure to qualified researchers upon reasonable request, subject to the approval of the corresponding data access committee/institution and compliance with applicable ethical and data protection requirements.

## Supplementary Materials

The following supporting information can be downloaded at https://www.mdpi.com/article/10.3390/sports14100458/s1, Table S1: PREFIS-AP randomized trial. SPIRIT 2025 checklist; Table S2: TIDieR checklist for the PREFIS-AP Exercise Prescription Program.

## Abbreviations

The following abbreviations are used in this manuscript:

6MWT	Six-Minute Walk Test
APISAL	Primary Care Research Unit of Salamanca
BMI	Body Mass Index
COPD	Chronic Obstructive Pulmonary Disease
FITT-VP	Frequency, Intensity, Time, Type, Volume, Progression
GPAQ	Global Physical Activity Questionnaire
IBSAL	Institute for Biomedical Research of Salamanca
ICH-GCP	International Council for Harmonization Good Clinical Practice
PREFIS-AP	Prescription of Physical Exercise in Primary Care
REDCap	Research Electronic Data Capture
SEEDO	Spanish Society for the Study of Obesity
SF-36	36-Item Short Form
TIDieR	Template for Intervention Description and Replication
WHO	World Health Organization
