# Supplementary material for: Effectiveness of a Physical Exercise Prescription Program in Primary Care to Improve Physical Fitness and Quality of Life in Adults Aged 65 Years and Older in a Rural Basic Health Area—Study Protocol for a Randomized Controlled Trial (PREFIS-AP)
Source: Sports (Basel). 2026 Oct 15;14(10):458. doi: 10.3390/sports14100458 (PMC13709371; doi:10.3390/sports14100458)
Supplement: Supplementary file 1 [file sports-14-00458-s001.zip › sports-4552466-supplementary.pdf]

Supplementary Materials

Table S1: PREFIS-AP randomized trial. SPIRIT 2025 checklist.

Section	Topic	Item	SPIRIT 2025 checklist item	Location in PREFIS-AP protocol
Administrative information	Title and structured summary	1a	Title stating the trial design, population, and interventions, with identification as a protocol	Title page
Administrative information	Title and structured summary	1b	Structured summary of trial design and methods, including items from the World Health Organization Trial Registration Data Set	Abstract; Trial registration statement; ClinicalTrials.gov record NCT07394790
Administrative information	Protocol version	2	Version date and identifier	Section 3.15, Trial status
Administrative information	Roles and responsibilities	3a	Names, affiliations, and roles of protocol contributors	Title page; Author Contributions
Administrative information	Roles and responsibilities	3b	Name and contact information for the trial sponsor	Funding statement; ClinicalTrials.gov record NCT07394790
Administrative information	Roles and responsibilities	3c	Role of trial sponsor and funders in design, conduct, analysis, and reporting of the trial, including any authority over these activities	Funding statement
Administrative information	Roles and responsibilities	3d	Composition, roles, and responsibilities of the coordinating site, steering committee, endpoint adjudication committee, data management team, and other individuals or	Sections 3.11 and 3.13; no separate trial committees are described

			groups overseeing the trial, if applicable	
**Section**	**Topic**	**Item**	**SPIRIT 2025 checklist item**	**Location in PREFIS-AP protocol**
Open science	Trial registration	4	Name of trial registry, identifying number (with URL), and date of registration. If not yet registered, name of intended registry	Abstract; Sections 3.1 and 3.15; ClinicalTrials.gov NCT07394790
Open science	Protocol and statistical analysis plan	5	Where the trial protocol and statistical analysis plan can be accessed	Section 3.12; finalized SAP deposited on Zenodo on 29 September 2026 (DOI: 10.5281/zenodo.23034729)
Open science	Data sharing	6	Where and how individual de-identified participant data (including data dictionary), statistical code, and any other materials will be accessible	Data Availability Statement
Open science	Funding and conflicts of interest	7a	Sources of funding and other support (e.g., supply of drugs)	Funding statement
Open science	Funding and conflicts of interest	7b	Financial and other conflicts of interest for principal investigators and steering committee members	Conflicts of Interest statement
Open science	Dissemination policy	8	Plans to communicate trial results to participants, healthcare professionals, the public, and other relevant groups (e.g., reporting in trial registry, plain-language summary, publication)	Section 3.14, Ethics and dissemination
**Section**	**Topic**	**Item**	**SPIRIT 2025 checklist item**	**Location in PREFIS-AP protocol**

Section	Topic	Item	SPIRIT 2025 checklist item	Location in PREFIS-AP protocol
Introduction	Background and rationale	9a	Scientific background and rationale, including summary of relevant studies (published and unpublished) examining benefits and harms for each intervention	Section 1, Introduction
Introduction	Background and rationale	9b	Explanation for choice of comparator	Sections 3.9 and 4
Introduction	Objectives	10	Specific objectives related to benefits and harms	Section 2, Objectives; Sections 3.10 and 3.13
Methods: patient and public involvement, trial design	Patient and public involvement	11	Details of, or plans for, patient or public involvement in the design, conduct, and reporting of the trial	Section 3.1; no formal patient or public involvement was planned
Methods: patient and public involvement, trial design	Trial design	12	Description of trial design, including type of trial (e.g., parallel group, crossover), allocation ratio, and framework (e.g., superiority, equivalence, non-inferiority, exploratory)	Section 3.1, Study design
Methods: participants, interventions, and outcomes	Trial setting	13	Settings (e.g., community, hospital) and locations (e.g., countries, sites) where the trial will be conducted	Section 3.2, Study setting
Methods: participants, interventions, and outcomes	Eligibility criteria	14a	Eligibility criteria for participants	Section 3.3, Participants and eligibility criteria
Methods: participants, interventions, and outcomes	Eligibility criteria	14b	If applicable, eligibility criteria for sites and for individuals who will deliver the interventions (e.g., surgeons, physiotherapists)	Section 3.8.3; no additional eligibility criteria for intervention providers were prespecified

Methods: participants, interventions, and outcomes	Intervention and comparator	15a	Intervention and comparator with sufficient details to allow replication, including how, when, and by whom they will be administered. If relevant, where additional materials describing the intervention and comparator can be accessed	Sections 3.8-3.9; Figure 1
Methods: participants, interventions, and outcomes	Intervention and comparator	15b	Criteria for discontinuing or modifying allocated intervention/comparator for a trial participant	Sections 3.8.1 and 3.13
Methods: participants, interventions, and outcomes	Intervention and comparator	15c	Strategies to improve adherence to intervention/comparator protocols, if applicable, and procedures for monitoring adherence	Section 3.8.3
Methods: participants, interventions, and outcomes	Intervention and comparator	15d	Concomitant care that is permitted or prohibited during the trial	Section 3.9, Control group
Methods: participants, interventions, and outcomes	Outcomes	16	Primary and secondary outcomes, including the specific measurement variable, analysis metric, method of aggregation, and time point for each outcome	Sections 3.6 and 3.10; Table 1; Figure 2
Methods: participants, interventions, and outcomes	Harms	17	How harms are defined and will be assessed (e.g., systematically, non-systematically)	Section 3.13, Safety monitoring and adverse events
Methods: participants, interventions, and outcomes	Participant timeline	18	Time schedule of enrolment, interventions (including any run-ins and washouts), assessments, and visits for participants. A schematic diagram is highly recommended	Figure 2; Sections 3.6 and 3.11

Section	Topic	Item	SPIRIT 2025 checklist item	Location in PREFIS-AP protocol
Methods: participants, interventions, and outcomes	Sample size	19	How sample size was determined, including all assumptions supporting the sample size calculation	Section 3.7, Sample size calculation
Methods: participants, interventions, and outcomes	Recruitment	20	Strategies for achieving adequate participant enrolment to reach target sample size	Section 3.4, Recruitment
Methods: assignment of interventions	Randomization: sequence generation	21a	Who will generate the random allocation sequence and the method used	Section 3.5, Randomization and allocation concealment
Methods: assignment of interventions	Randomization: sequence generation	21b	Type of randomization (simple or restricted) and details of any factors for stratification; other restriction details should be unavailable to those who enrol participants or assign interventions	Section 3.5; simple unrestricted randomization without blocking or stratification
Methods: assignment of interventions	Allocation concealment mechanism	22	Mechanism used to implement the random allocation sequence, describing steps to conceal the sequence until interventions are assigned	Section 3.5
Methods: assignment of interventions	Implementation	23	Whether personnel who enrol and those who assign participants to interventions will have access to the random allocation sequence	Section 3.5
Methods: assignment of interventions	Blinding	24a	Who will be blinded after assignment to interventions (e.g., participants, care providers, outcome assessors, data analysts)	Section 3.5; Section 4, Discussion

Section	Topic	Item	SPIRIT 2025 checklist item	Location in PREFIS-AP protocol
Methods: assignment of interventions	Blinding	24b	If blinded, how blinding will be achieved and description of the similarity of interventions	Not applicable to participants and intervention providers because of the behavioural intervention; coded group labels for the primary statistical analysis are described in Section 3.5
Methods: assignment of interventions	Blinding	24c	If blinded, circumstances under which unblinding is permissible and procedure for revealing a participant's allocated intervention during the trial	Not applicable; participants and intervention providers are not blinded
Methods: data collection, management, and analysis	Data collection methods	25a	Plans for assessment and collection of trial data, including processes to promote data quality, and description of trial instruments with reliability and validity if known; reference to accessible data collection forms if not in the protocol	Sections 3.6–3.6.6 and 3.11; Table 1
Methods: data collection, management, and analysis	Data collection methods	25b	Plans to promote participant retention and complete follow-up, including outcome data to be collected for participants who discontinue or deviate from intervention protocols	Sections 3.8.3 and 3.13
Methods: data collection, management, and analysis	Data management	26	Plans for data entry, coding, security, and storage, including processes to promote data quality; reference to accessible data management procedures if not in the protocol	Section 3.11, Data collection and management

Section	Topic	Item	SPIRIT 2025 checklist item	Location in PREFIS-AP protocol
Methods: data collection, management, and analysis	Statistical methods	27a	Statistical methods used to compare groups for primary and secondary outcomes, including harms	Section 3.12, Statistical analysis
Methods: data collection, management, and analysis	Statistical methods	27b	Definition of who will be included in each analysis and in which group	Section 3.12 (intention-to-treat; participants analysed as randomized)
Methods: data collection, management, and analysis	Statistical methods	27c	How missing data will be handled in the analysis	Section 3.12
Methods: data collection, management, and analysis	Statistical methods	27d	Methods for any additional analyses (e.g., subgroup and sensitivity analyses)	Section 3.12
Methods: monitoring	Data monitoring committee	28a	Composition of the data monitoring committee (DMC), summary of its role and reporting structure, independence, conflicts of interest, and reference to its charter; alternatively, explanation of why a DMC is not needed	Section 3.13; a separate DMC is not planned because the intervention is short, non-pharmacological, and considered low risk, with direct medical oversight
Methods: monitoring	Data monitoring committee	28b	Explanation of interim analyses and stopping guidelines, including who will have access to interim results and make the final decision to terminate the trial	Section 3.12 states that no interim efficacy analysis or formal statistical stopping rule is planned
Methods: monitoring	Trial monitoring	29	Frequency and procedures for monitoring trial conduct; if there is no monitoring, give an explanation	Sections 3.11 and 3.13; no separate external trial-monitoring process was planned
Ethics	Research ethics approval	30	Plans for seeking research ethics committee/institutional review board approval	Section 3.14; Institutional Review Board Statement

Section	Topic	Item	SPIRIT 2025 checklist item	Location in PREFIS-AP protocol
Ethics	Protocol amendments	31	Plans for communicating important protocol modifications to relevant parties	Section 3.14
Ethics	Consent or assent	32a	Who will obtain informed consent or assent from potential participants or authorized proxies, and how	Sections 3.3, 3.4, and 3.14; informed consent will be obtained by a member of the research team before any study-specific procedure
Ethics	Consent or assent	32b	Additional consent provisions for collection and use of participant data and biological specimens in ancillary studies, if applicable	Not applicable; no ancillary studies requiring additional consent for the collection or use of participant data or biological specimens were planned
Ethics	Confidentiality	33	How personal information about potential and enrolled participants will be collected, shared, and maintained to protect confidentiality before, during, and after the trial	Sections 3.11 and 3.14; Data Availability Statement
Ethics	Ancillary and post-trial care	34	Provisions, if any, for ancillary and post-trial care and for compensation to those who suffer harm from trial participation	Section 3.13; participants retain access to routine clinical and emergency care. No additional study-specific post-trial care or compensation arrangements were planned

Source and attribution: Chan A-W, Boutron I, Hopewell S, Moher D, Schulz KF, et al. SPIRIT 2025 statement: updated guideline for protocols of randomised trials. BMJ. 2025;389:e081477. doi:10.1136/bmj-2024-081477. Checklist text reproduced under the Creative Commons Attribution 4.0 International License. Official resources.

Table S2: TIDieR checklist for the PREFIS-AP Exercise Prescription Program.

TIDieR item	Item	Description of the PREFIS-AP intervention
1	**Brief name**	**PREFIS-AP individualized multicomponent Exercise Prescription Programme**, a 12-week exercise intervention integrated into routine Primary Care and supported by the MEDORA® electronic health record decision-support system.
2	**Why**	The intervention was designed to translate clinical and functional assessment into an individualized exercise prescription rather than providing generic physical-activity advice. The prescription framework considers exercise as a therapeutic intervention requiring specification of type, intensity, duration, frequency and progression according to the participant's clinical and functional status.
3	**What: materials**	The intervention will use the exercise prescription module built into MEDORA® along with an individualized exercise prescription framework based on the methodology of the Regional Center for Sports Medicine of Castilla y León (CEREMEDE). The clinical prescription framework and the allowed ranges are described in this checklist; the complete internal decision logic of the MEDORA® module is not publicly available.
4	**What: procedures**	Before receiving a prescription, participants will go through clinical and functional check-ups. The information gathered will be used to adjust the type, intensity, duration, frequency, and progression of exercises for each person. The program will combine aerobic, strength, balance, and flexibility exercises, adding breathing exercises when needed. Aerobic workouts can be continuous, intermittent, or interval-based if deemed appropriate. Strength exercises will focus on the main muscle groups, with intensity adjusted to each person's ability. Exercise plans will be reinforced and adjusted according to participants' tolerance and progress.
5	**Who provided**	The prescription of individualized exercise is integrated into Primary Care. Clinical evaluation and prescription will be done within the Primary Care team, while supervised exercise and follow-up will be provided by the exercise professional involved in the program. The exercise professional will oversee participants during group follow-up sessions, while each participant will follow their own individualized prescription.
6	**How**	The exercise will be prescribed individually and done independently at home and during supervised group sessions. During the group sessions, participants will follow individualized instructions instead of a uniform group program.
7	**Where**	Home-based exercise and supervised group sessions in municipal sports facilities within the rural Basic Health Area.
8	**When and how much**	The intervention will last 12 weeks. There won't be a single initial dose that applies to all participants; the starting prescription will be personalized based on the baseline clinical and functional assessment within a predefined exercise prescription framework. Generally, aerobic exercise will be prescribed at least 3 days a week, with 4–5 days a week as the target frequency; continuous aerobic sessions will usually last between 30 and 60 minutes, depending on each participant's fitness level. The intensity of the aerobic exercise will be tailored using heart rate–based goals and/or perceived exertion (Borg scale). Resistance exercises will target the major muscle groups and will be prescribed 2–3 non-consecutive days per week, progressing based on initial capacity and tolerance. The framework will include 10 to 15 repetitions, progressing up to a maximum of 3 sets and a perceived exertion around OMNI-RES 5–7, adjusting the resistance accordingly. Warm-up and cool-down periods

		will be included. There will be supervised group strength sessions three days a week. Breathing exercises will only be included when there is a clinical indication; specific eligibility criteria or a standardized dose for this component will not be defined in advance.
**TIDieR item**	**Item**	**Description of the PREFIS-AP intervention**
**9**	**Tailoring**	Individualization will be a key feature of the intervention. The type and dose of exercise will be selected based on the initial clinical condition, fitness level, functional capacity, previous physical activity, tolerance, and individual circumstances. Progression will be gradual and based on the participant's response. For aerobic exercise, duration can be increased before intensity, and participants may move from intermittent to continuous exercises and, when considered appropriate, to interval exercises. Resistance exercise will be progressed by adjusting repetitions, sets, and/or resistance according to tolerance and perceived effort.
**10**	**Modifications**	The recipe can be modified during the 12-week intervention based on clinical status, exercise tolerance, functional progression, and difficulties encountered while following the prescribed program. The booster sessions will provide an opportunity to review performance in the exercises and adjust the individualized program when necessary. No uniform changes will be applied to all participants.
**11**	**How well: planned**	The fidelity of the intervention will be supported by an initial standard assessment, individualized prescription within the framework backed by MEDORA®, instruction to the participants, supervised booster sessions, and follow-up of the prescribed program. Adherence and any relevant modifications or deviations from the prescribed program will be recorded according to the study procedures.
**12**	**How well: actual**	As this manuscript reports the study protocol, comparative intervention-effect results are not presented. Actual adherence, implementation, modifications and deviations from the prescribed intervention will be reported with the trial results.
**Abbreviations:** MEDORA®, electronic health record system of the Regional Health Service of Castilla y León; OMNI-RES, OMNI Resistance Exercise Scale; PREFIS-AP, Physical Exercise Prescription in Primary Care trial; TIDieR, Template for Intervention Description and Replication.		
